# Using oral molecular hydrogen supplements to combat microinflammation in humans: a pilot observational study

**DOI:** 10.7150/ijms.101114

**Published:** 2024-09-09

**Authors:** Kuo-Cheng Lu, Min-Chung Shen, Reui-Lin Wang, Wen-Wen Chen, Szu-Han Chiu, Yung-His Kao, Feng-Cheng Liu, Po-Jen Hsiao

**Affiliations:** 1 Division of Nephrology, Department of Internal Medicine, Taipei Tzu Chi Hospital, Buddhist Tzu Chi Medical Foundation, New Taipei City, Taiwan.; 2 Division of Nephrology, Department of Internal Medicine, Tri-Service General Hospital, National Defense Medical Center, Taipei, Taiwan.; 3 Division of Nephrology, Department of Internal Medicine, Fu-Jen Catholic University Hospital, School of Medicine, Fu-Jen Catholic University, New Taipei City, Taiwan.; 4 Rheumatology/Immunology and Allergy, Department of Medicine, Armed Forces Taoyuan General Hospital, Taoyuan, Taiwan.; 5 Division of Medicine, Armed Forces Taoyuan General Hospital, Taoyuan, Taiwan.; 6 Nursing Department, Min-Sheng General Hospital, Taoyuan, Taiwan.; 7 Division of Endocrinology and Metabolism, Department of Medicine, Armed Forces Taoyuan General Hospital, Taoyuan, Taiwan.; 8 Department of Life Sciences, National Central University, Taoyuan, Taiwan.; 9 Rheumatology/Immunology and Allergy, Department of Medicine, Tri-Service General Hospital, National Defense Medical Center, Taipei, Taiwan.; 10 Division of Nephrology, Department of Internal Medicine, Taoyuan Armed Forces General Hospital, Taoyuan, Taiwan.

**Keywords:** antioxidant, anti-inflammatory effects, chronic diseases, chronic inflammation, molecular hydrogen, oral solid hydrogen capsules (OSHCs)

## Abstract

**Background:** Persistent inflammation over time can cause gradual harm to the body. Molecular hydrogen has the potential to specifically counteract reactive oxygen species (ROS), reduce disease severity, and enhance overall health. Investigations of the anti-inflammatory and antioxidant properties of oral solid hydrogen capsules (OSHCs) are currently limited, prompting our examination of the beneficial effects of OSHCs. Subsequently, we conducted a clinical study to assess the impact of OSHCs supplementation on individuals with chronic inflammation.

**Materials and methods:** Initially, we evaluated the oxidative reduction potential (ORP) properties of the OSHCs solution by comparing it to hydrogen-rich water (HRW) and calcium hydride (CaH_2_) treated water. In our outpatient department, stable patients with chronic illnesses who were treated with varying doses of OSHCs were randomized into low-, medium-, and high-dose groups for 4 weeks. Primary outcomes included changes in the serum erythrocyte sedimentation rate (ESR) and C-reactive protein (CRP) concentrations after four weeks of OSHCs consumption. Secondary outcomes included changes in the Brief Fatigue Inventory-Taiwan (BFI-T) fatigue scale, Control Status Scale for Diabetes (CSSD70) scores, and Disease Activity Score 28 (DAS28).

**Results:** Compared to HRW and CaH_2_, OSHCs demonstrated a prolonged reduction in ORP for 60 minutes in vitro and enabled a regulated release of hydrogen over 24 hours. A total of 30 participants, with 10 in each dosage (low/medium/high) group, completed the study. The average ESR120 significantly decreased from the first week to the fourth week, with a noticeable dose effect (low-dose group, p = 0.494; high-dose group, p = 0.016). Overall, the average CRP concentration showed a distinct decreasing trend after four weeks of OSHCs administration (w0 vs. w4, p = 0.077). The average DAS28 score demonstrated a significant decrease following OSHCs treatment. Furthermore, there were improvements in the BFI-T and CSSD70 scores.

**Conclusion:** OSHCs supplementation may exert anti-inflammatory and antioxidant effects on individuals with chronic inflammation. However, further clinical studies could be investigated to explore the potential therapeutic effects of OSHCs.

## Introduction

Inflammation is the natural immune defense of the body against foreign pathogens. Prolonged immune system activation leads to chronic inflammation and a persistent systemic response involving continuous cytokine release. Persistent chronic inflammation can result in cumulative damage to the human body, contributing to the development of various chronic diseases, including cardiovascular disease, brain damage, cancer, rheumatoid arthritis, asthma, diabetes, metabolic disorders, and dementia, as indicated by recent studies 1-6. The persistent release of proinflammatory cytokines from immune-related cells and activation of the innate immune system can lead to chronic systemic inflammation, thereby playing a role in the development or progression of chronic medical conditions. C-reactive protein (CRP) and the erythrocyte sedimentation rate (ESR) serve as clinical markers to aid physicians in the accurate diagnosis and monitoring of various acute and chronic conditions 4-6. Treating the intricate clinical manifestations of chronic inflammation remains challenging, and no effective treatments are currently available.

Recent research highlights that employing molecular hydrogen as an antioxidant effectively improves symptoms associated with chronic inflammation-related diseases 7. Molecular hydrogen has notable effects on cellular oxygen radicals. It selectively reduces highly toxic oxygen radicals such as hydroxyl radicals and peroxynitrite, demonstrating a protective role against oxidative stress 8. In addition, the present data suggest that the cellular protective effects facilitated by molecular hydrogen can be attributed to the adjustment of cellular antioxidant defenses, including antioxidant and cytoprotective genes, along with intracellular and extracellular redox signaling 9. Furthermore, hydrogen interferes with reactive oxygen species (ROS) in living systems and serves as an antioxidant by selectively reducing cytotoxic oxygen radicals 10. While the antioxidant hypothesis posits that the scavenging of hydroxyl radicals can partially explain certain biological effects of H_2_, the long debate surrounding H_2_ status stems from the significantly lower reaction rate of H_2_ with hydroxyl radicals than with other cellular antioxidants. In addition to its antioxidant function, H_2_ has been shown to have various effects, including anti-inflammatory, antiapoptotic, antishock, antidysmetabolic, and autophagy-activating effects and the ability to preserve mitochondrial function 8, 11. In summary, supplementation with molecular hydrogen eliminates the damage caused by malignant free radicals while also eliminating chronic inflammation. For example, in patients with Parkinson's disease 9, cardiovascular disease 9, rheumatoid arthritis 12, allergic rhinitis 13, 14, atopic dermatitis 15, 16, asthma 17, 18, chronic obstructive pulmonary disease 19, and other diseases, hydrogen supplementation has been shown to reduce inflammation and improve symptoms.

There have been an increasing number of clinical studies on molecular hydrogen, such as nasal inhalation of hydrogen, injection of hydrogen saline, and drinking of hydrogen-rich water (HRW). A recent human study revealed that gaseous hydrogen reduces lung damage from cigarette poisoning 20. Our recent article also demonstrates that molecular hydrogen not only scavenges harmful oxygen radicals but also promotes the production of antioxidant enzymes through various redox signaling pathways 21. However, studies on solid dosage forms of molecular hydrogen have been limited to animal experiments 22. We performed an observational study focusing on the use of oral molecular hydrogen supplements in humans. This study aimed to evaluate the effect of oral solid hydrogen capsules (OSHCs) supplementation on the inflammatory status of patients with chronic inflammation through scientific assessments and objective clinical indicators.

## Materials and methods

### Study design

In a fundamental investigation, we explored the antioxidant effects of OSHCs by contrasting them with those of HRW and calcium hydride (CaH_2_). To investigate the antioxidant capacity of OSHCs, calcium hydride, and HRW, we measured the reduction potential of these different solutions using a reduction potential detector (Pen type oxidative reduction potential (ORP) Meter, ORP5041). The greater the negative value of the reduction potential, the stronger the antioxidant capacity. The longer the negative value of reduction potential lasts, the longer the anti-oxidation time. For the OSHCs and CaH_2_, 1 g of each was added to 100 ml of pure water and stirred with a glass rod for 3 minutes. The hydrogen-rich water (HRW) was obtained through electrolysis, resulting in a hydrogen concentration of 1,200 ppb.

**Figure 1:**
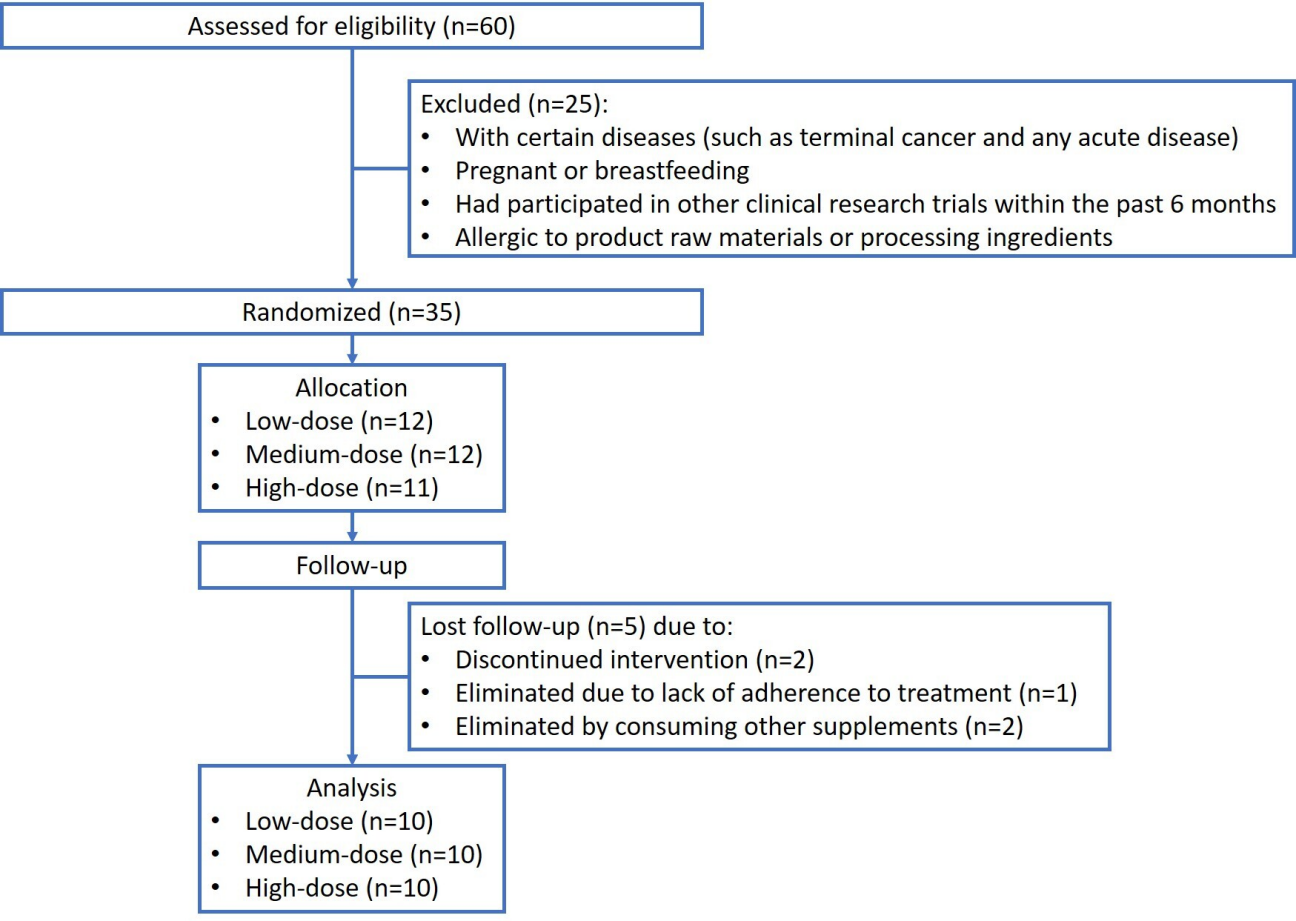
Study flowchart.

In the clinical study, participants were selected from the hospital's general medicine outpatient department. The screening criteria included adults aged 20 years or older with chronic stable conditions, such as diabetes, hypertension, and metabolic syndrome. Regarding recruitment, researchers followed up with chronic patients with recurring outpatient visits for more than three months before enrolment. During outpatient visits, the researchers explained the content and procedure of the study, including patient cooperation, to the patients. The researchers ensured that each patient understood the entire study process and signed an informed consent form. This clinical study was performed in July 2022 (Figure 1).

Patients who had unstable or advanced medical diseases (such as acute illness or terminal cancer), who were pregnant or breastfeeding, who had participated in other clinical research trials within the past 6 months, or who were allergic to the raw materials or processing ingredients in the supplements were excluded. None of these stable patients had any adjustment to their medications in the outpatient setting. The main outcomes assessed were changes in the blood ESR (120) and serum CRP concentrations. In addition, the Disease Activity Score 28 (DAS28) is a metric used to assess disease activity in rheumatoid arthritis (RA). "DAS" stands for "disease activity score," and the number 28 refers to the 28 joints evaluated in this measurement.

Secondary outcomes included changes in scores on the Brief Fatigue Inventory-Taiwan (BFI-T) fatigue scale, the Control Status Scale for Diabetes (CSSD70) questionnaire, and the DAS28.

### Research method: Antioxidant hydrogen supplements

The OSHCs were integrated into the existing treatment regimen without altering other prescriptions and while maintaining the same dosage and frequency of clinical treatment. The original methods for coral calcium-carrying hydrogen have been described 23, 24*.* The OSHCs were provided in solid dosage form and used as adjuvants every day.

- Low-dose: 1 capsule per day (in the morning on an empty stomach; 1 capsule × 28 days = 28 capsules)
- Medium dose: 3 capsules per day (in the morning on an empty stomach; 3 capsules × 28 days = 84 capsules)
- High-dose: 6 capsules per day (3 capsules in the morning and 3 capsules in the evening; 6 capsules × 28 days = 168 capsules)

The patients were instructed to take the capsules for 4 weeks and were informed that they could leave the study at any time.

***Sample size.*** The anti-inflammatory effects of OSHCs supplementation in patients with chronic diseases were the primary outcome. In this study, it was assumed that based on a 10% improvement in anti-inflammatory effects with a power of 90% and an alpha value of 0.05, a sample size of 30 patients was needed.

### Materials

**Figure 2:**
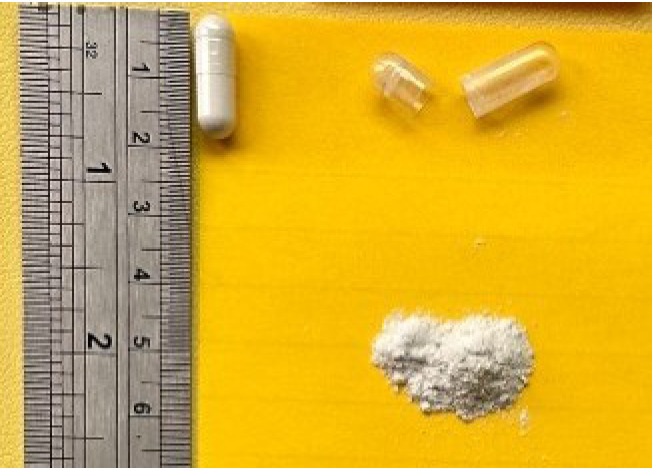
Demonstration of OSHCs, purchased from HOHO Biotech Co., Ltd. (Taipei, Taiwan).

The OSHCs (hydrogen-rich coral calcium) were purchased from HOHO Biotech Co., Ltd. (Taipei, Taiwan; Figure 2). Each capsule contained 0.17 mg of hydrogen-rich coral calcium, and each gram of capsule contained 4.4 × 10^20 molecules of hydrogen, equivalent to 7.5 × 10^16 molecules of hydrogen per capsule. Coral calcium infused with hydrogen is a safe and convenient source of hydrogen. The original method for coral calcium-carrying hydrogen has been described previously. The coral powder with calcium carbonate was treated by circulating 100% H_2_ gas at 800°C for 1 h (high temperature) and at 300°C for 4 h (low temperature) to obtain hydrogen powder with a 10 μm particle size through grinding.

### Measurements of adverse effects/symptoms

During the four weeks, each participant consumed the prescribed capsules. Upon their subsequent visit to the hospital, 20 ml of blood and 10 ml of urine were obtained. Additionally, body weight, blood pressure, and blood oxygen levels were evaluated. Adverse reactions were meticulously documented, and the participants completed a health assessment questionnaire.

### Statistical analysis

Statistical analyses were conducted using R statistical software (version 4.0.4; R Core Team 2021) on a Windows platform. The results are presented as the mean or interquartile range (IQR). To compare the values before and after the use of OSHCs for each group, a paired t-test was used. To compare age, the ESR120, CRP concentrations, and the BFI-T-and CSSD70 scores among the three groups, one-way ANOVA was employed, and the chi-square test was used to compare the sex distribution of patients in different groups. The data are expressed as the means ± standard deviations (means ± SDs) along with 95% confidence intervals (CIs). A p-value <0.05 was considered to indicate statistical significance.

## Results

### Comparison of the reduction potential of OSHCs, HRW, and CaH_2_

**Figure 3:**
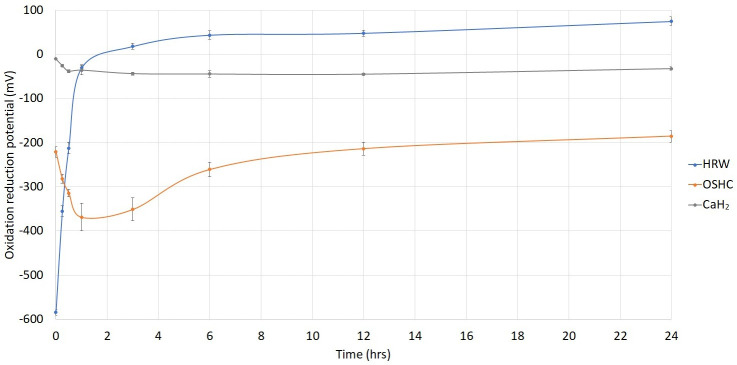
The use of oral OSHCs provides advantages over hydrogen-rich water (HRW) and calcium hydride (CaH_2_) due to their stable and sustained strong reduction potential.

In contrast to the limited 60-minute decrease reduction potential (antioxidative effect) of the HRW solution, the OSHCs solution enabled controlled hydrogen release over 24 hours with a sustained strong reduction potential. The higher the reduction potential value, the OSHCs solution demonstrated superior persistent pharmacological effects and greater stability in terms of reduction potential compared to the CaH_2_ and HRW solutions. In vitro experiments confirmed a 24-hour sustained antioxidant effect, as the reduction potential of the OSHCs persisted for the same duration (Figure 3).

**Figure 4:**
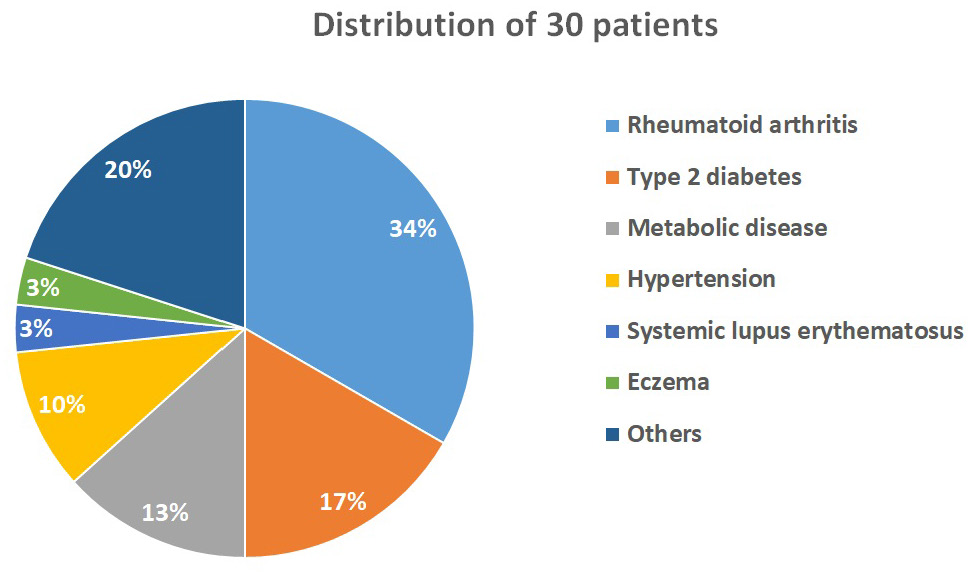
Chronic illness and distribution of all patients.

***Demographics.*** A total of 30 patients were randomly assigned to 3 10-person groups (low-, medium-, and high-dose groups) and completed the study. The participants included 10 males and 20 females, with a mean age of 64.86 years (range: 32-82 years; Table 1). Notably, none of the patients who underwent OSHCs supplementation reported adverse effects or toxicity. All participants had preexisting chronic illnesses and were in a state of chronic inflammation. Among the cohort, approximately one-third of the participants had rheumatoid arthritis (n=10), five had type 2 diabetes mellitus, four had metabolic diseases, and three had hypertension (Figure 4).

### The primary outcome measures focused on alterations in inflammatory markers (the ESR120 and CRP concentrations)

This study aimed to assess the antioxidative effects of OSHCs supplementation as an adjuvant treatment. Thirty participants were involved in the study over 4 weeks. Blood and urine test results, along with diagnostic scale scores, were evaluated at three time points: before capsule intake (w0), one-week post-capsule intake (w1), and four weeks post-capsule intake (w4). The participants were randomly allocated into three groups—low-, medium-, and high-dose—to investigate dose-related effects.

### Changes in the ESR120

**Figure 5:**
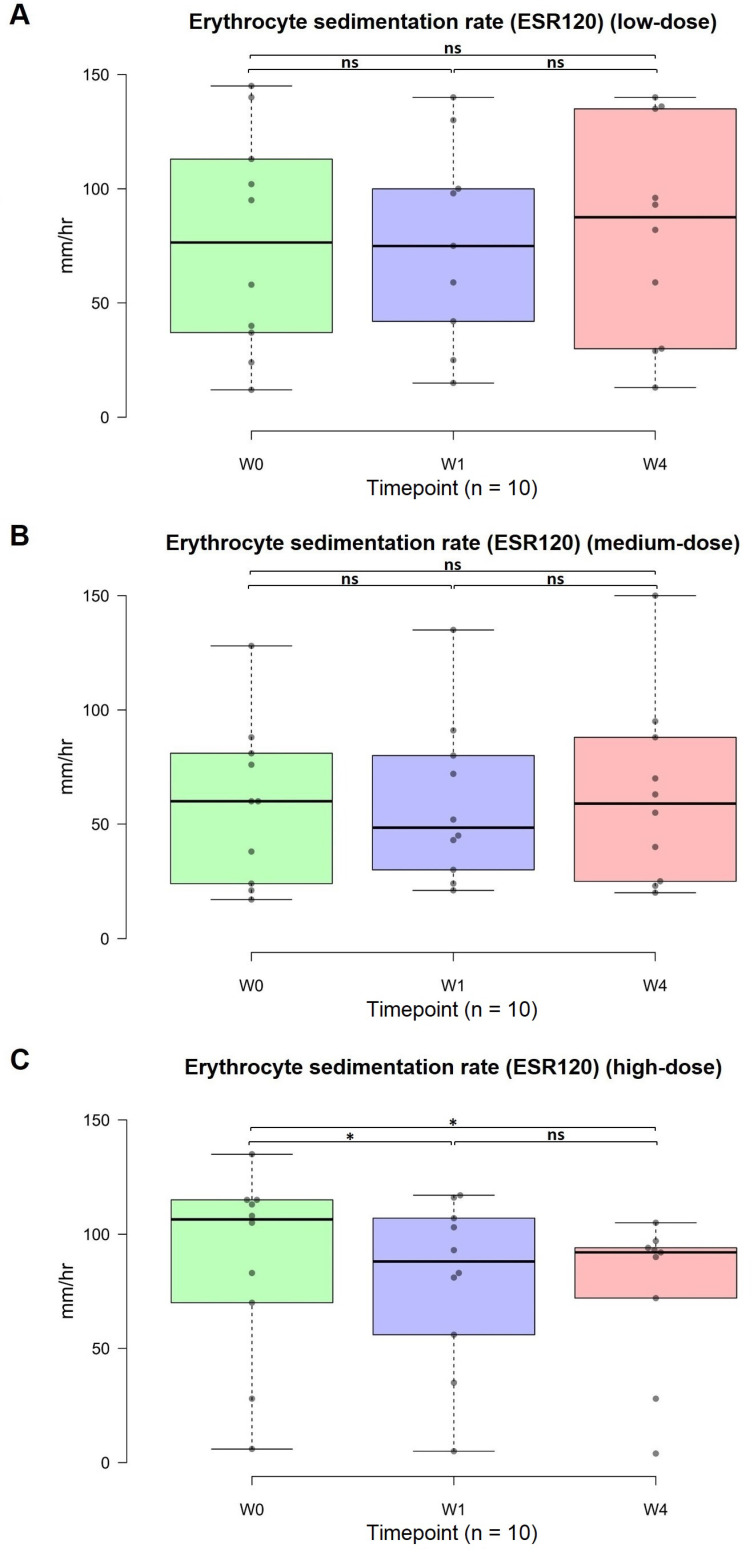
Changes in the erythrocyte sedimentation rate (ESR)-120 from 0 to 4 weeks (n=30). Normal range: males, <10; females, <15 mm/hr. (A) Low-dose group. (B) Medium-dose group. (C) High-dose group. The data are represented by boxplots (ns: not significant; *p < 0.05; **p < 0.01; ***p < 0.001) and were analyzed using paired t-tests.

The standard value for the ESR120 is 0-20 mm/h. Notably, in the high-dose group, the anti-inflammatory response exhibited a rapid onset, achieving significant effects in the first week and further improvement in the fourth week. Specifically, the mean ESR120 in the high-dose group decreased from an average of 87.8 to 79.6 after the first week (w0 vs. w1, p = 0.034, 95% CI: 56.66, 102.54) to 75 after the fourth week (w0 vs. w4, p = 0.016, 95% CI: 53.26, 96.74). Although no significant differences were detected between the low-dose (p = 0.494, 95% CI: 51.93, 110.67; Figure 5A) and medium-dose (p = 0.195, 95% CI: 37.81, 87.99; Figure 5B) groups, a significant dose-dependent difference was detected in the high-dose group (p = 0.016, 95% CI: 53.26, 96.74; Figure 5C). Further clarification is warranted to determine whether extending the administration of OSHCs from four weeks to three or six months at low and medium doses could lead to improved anti-inflammatory effects.

### Changes in CRP concentrations

**Figure 6:**
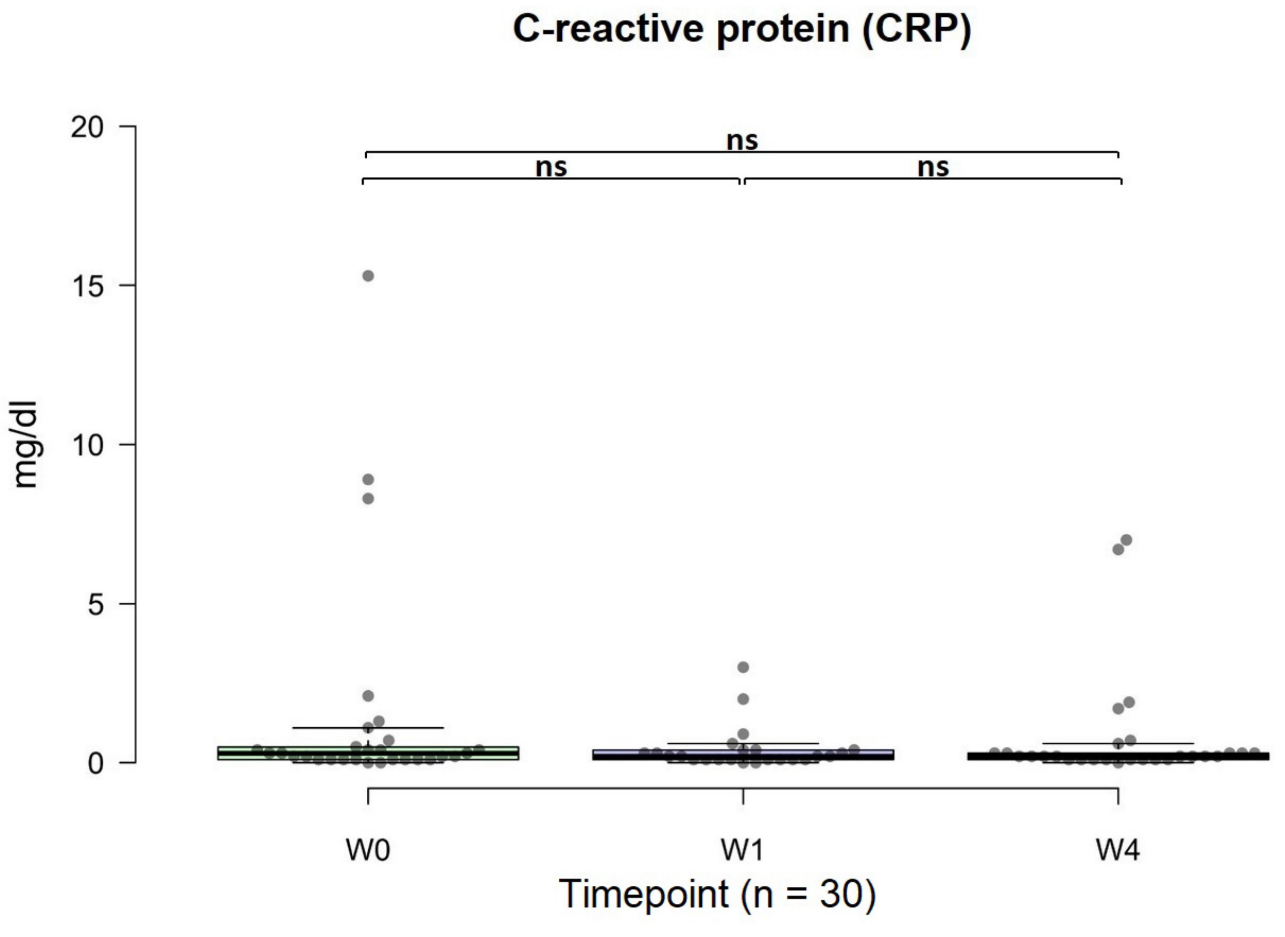
Changes in C-reactive protein (CRP) from 0 to 4 weeks (n=30). The normal range was < 1 mg/dl. The data are represented by boxplots (ns: not significant; *p < 0.05; **p < 0.01; ***p < 0.001) and were analyzed using paired t-tests.

The standard CRP concentration was <1 mg/dl. In general, there was an anti-inflammatory effect on CRP concentrations, irrespective of dosage. The average CRP level before OSHCs usage was 1.46 mg/dl, and after four weeks of use, the average CRP level was 0.80 mg/dl, meeting the criterion of <1 mg/dl, with no distinction based on dosage. Although not statistically significant, there was an observable anti-inflammatory trend (w0 vs. w4, p = 0.077, 95% CI: -0.29, 1.90) (Figure 6).

### The secondary outcome measures were the changes in the BFI-T, CSSD70, and DAS28 scores

**Figure 7:**
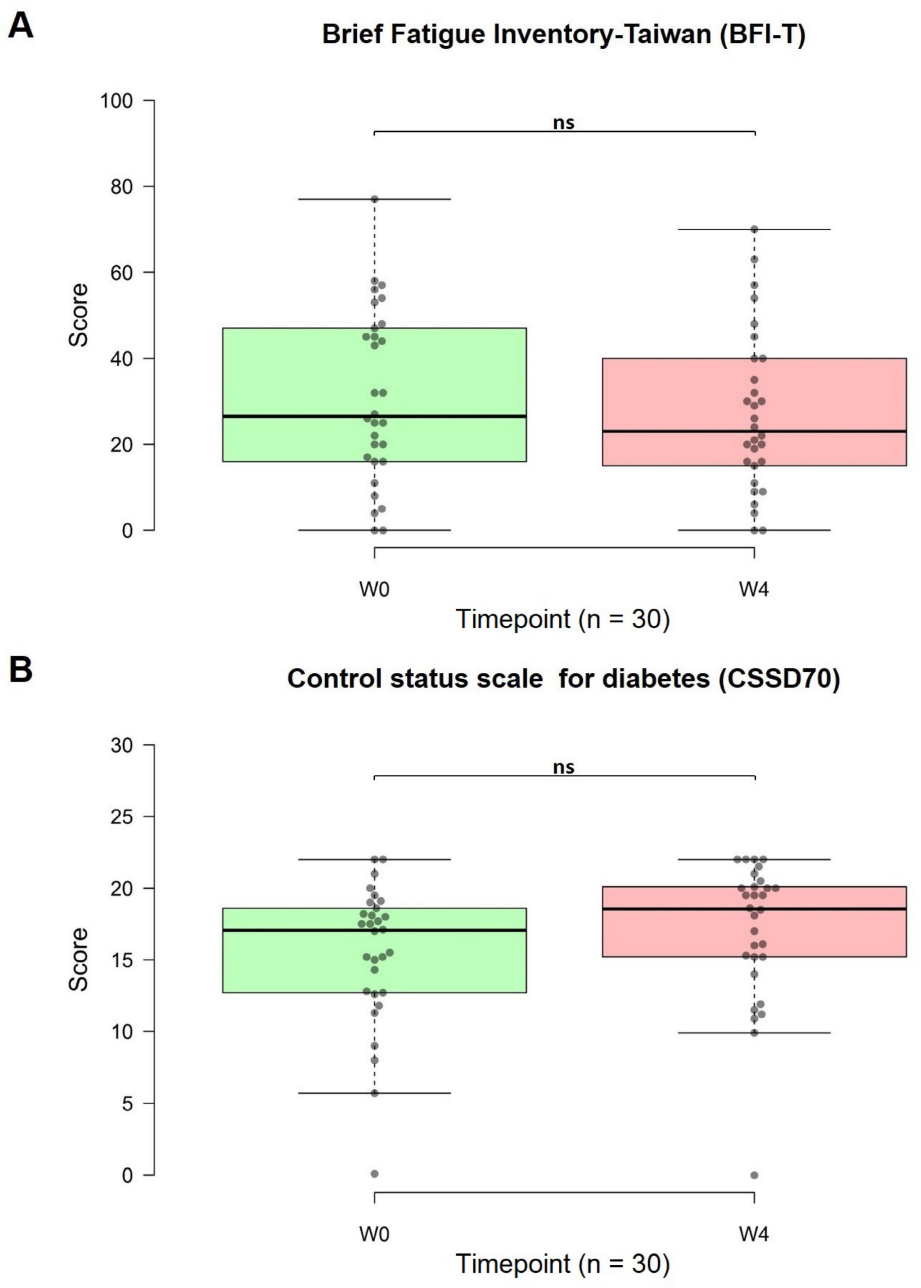
Changes in the Brief Fatigue Inventory-Taiwan (BFI-T) and Control Status Scale for Diabetes (CSSD70) scores from before to after treatment (n=30). (A) The BFI-T consists of 10 questions, each scored from 0 to 10, with 0 = most healthy and 10 = most severe (total range: 0 to 100). (B) The CSSD70 questionnaire consists of 11 questions, each scored from 0 to 2 (total range: 0 to 22). The data are represented by boxplots (ns: not significant; *p < 0.05; **p < 0.01; ***p < 0.001) and were analyzed using paired t-tests.

Eight out of 30 patients experienced improved sleep quality, while seven out of 30 patients reported increased energy levels. Lower BFI-T scores indicated reduced fatigue. The use of OSHCs for one month improved chronic fatigue. The average score before capsule consumption was 31.10, and after four weeks of use, the average score decreased to 27.03. Although the difference was not statistically significant, a noticeable downward trend was observed (p = 0.125, 95% CI: 15.56, 38.51; Figure 7A).

Higher CSSD70 scores indicate improved diabetes control. After one month of OSHC use, the CSSD70 scores increased. The average CSSD70 score before OSHCs consumption was 15.38, which increased to 16.97 after four weeks of use. Although the difference was not statistically significant, there was an observable upward trend (p = 0.063, 95% CI: 13.93, 20.00; Figure 7B). Extending the duration of use or expanding the sample size may have resulted in statistically significant differences.

**Figure 8:**
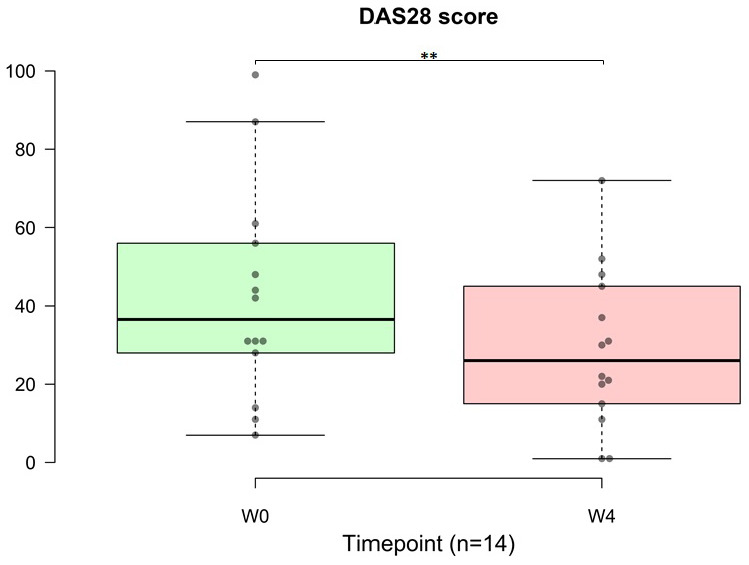
Distribution of patients and changes in the Disease Activity Score in 28 joints (DAS28) from before to after treatment (n=14). (A) Distribution of all 30 patients. There were 14 patients with autoimmune diseases, accounting for nearly half of all patients (47%). (B) The DAS28 is a measure of disease activity in autoimmune diseases. The data are represented by boxplots (ns: not significant; *p < 0.05; **p < 0.01; ***p < 0.001) and were analyzed using paired t-tests.

Among the entire cohort of 30 patients, 14 (47%) were diagnosed with autoimmune disease. In the context of the DAS28, a decrease in the score signifies an improvement in disease activity in patients with autoimmune conditions. Notably, one month of OSHCs supplementation resulted in a significant improvement in DAS28 score in 14 patients with autoimmune diseases (p = 0.002, 95% CI: 18.38, 39.62; Figure 8). The average DAS28 score recorded before OSHC consumption was 42.14, and after four weeks of use, it decreased to 29.00.

## Discussion

Chronic inflammation poses a substantial and escalating burden to the global healthcare system. The prevailing consensus is that a high intake of dietary bioactive compounds, particularly nutritional supplements, may be linked to a reduced risk of chronic illnesses. Nutritional supplements, including oral molecular hydrogen, have the potential to aid in the management of chronic diseases by diminishing oxidative stress and the associated inflammatory pathways. Our findings revealed that OSHCs supplementation exerts a significant dose-dependent anti-inflammatory effect on chronic inflammation in the human body. Importantly, none of the patients exhibited adverse reactions, and no patients withdrew from the study. These results effectively underscore the potential of OSHCs supplementation to mitigate chronic inflammation in humans, providing optimism for its future application as a new adjunct in clinical treatments.

### Bioengineering and clinical application of molecular hydrogen

As the smallest molecule in nature, hydrogen can readily diffuse into organs and tissues of the human body. In 2007, Professor Ohsawa recommended the use of hydrogen gas as a therapeutic antioxidant, emphasizing its ability to selectively reduce cytotoxic oxygen radicals 7. Initially, molecular hydrogen functions as a mild antioxidant, eliminating toxic free radicals without disrupting the body's redox balance. After 16 years of extensive animal and human experiments, the antioxidant and anti-inflammatory effects, along with the mechanism of action of molecular hydrogen, have been elucidated in numerous supporting studies. Despite repeated validation of its beneficial effects and the absence of biological toxicity in thousands of studies and dozens of clinical trials over 16 years, molecular hydrogen has not been widely applied as a medical treatment in hospitals. The primary obstacle is the low clinical availability of this technology. For instance, HRW has a low hydrogen content, and molecular hydrogen volatilizes quickly, resulting in a lower effective concentration in HRW. In addition, hydrogen inhalation and HRW machines are expensive, bulky, and complex to operate, making it challenging to determine the appropriate dosage. Consequently, large-scale human trials are challenging to conduct. To address these clinical usability issues, there is a need for a new technology that delivers an adequate dose of hydrogen, is portable and user-friendly, and allows precise dosage control.

OSHCs offer a solution to the limitations associated with hydrogen inhalation and HRW. In terms of pharmacology, dose adjustments, and clinical availability, OSHCs have advantages over earlier technologies, such as HRW and CaH_2_, as they provide controlled release and improved stability (Figure 3).

1. The stability of the reduction potential is notable, featuring 24-hour controlled release characteristics. In contrast to HRW, which has an antioxidative effect lasting only 60 min, OSHCs ensure the controlled release of hydrogen over 24 hours. This resulted in favorable pharmacokinetic effects, offering improved stability of the reduction potential. In vitro experiments confirmed the 24-hour antioxidant effects, confirming that the anti-inflammatory effects of OSHCs also extended for 24 h.

2. Convenient dosage adjustment is facilitated, allowing for precise customization based on individual patient needs and conditions. Patients with varying indications and weights can take one, three, or six tablets per day. The established safe dose is up to 13 tablets daily, exceeding which is not toxic; however, surpassing this limit would result in calcium intake surpassing the recommended daily limit set by the Taiwan Food and Drug Administration (TFDA) (https://consumer.fda.gov.tw/Food/CapsuleAuditQueryDetail.aspx?nodeID=165&id=1096000144).

3. Optimal clinical usability is achieved by avoiding bulky high-pressure steel cylinders or machines, which are typically necessary for producing HRW. Such equipment is expensive, intricate, and inconvenient to operate.

To measure molecular hydrogen concentration in an aqueous solution, methods differ in terms of sensitivity, accuracy, and available equipment. Gas chromatography is one such method, which analyzes gas samples from the solution using either a thermal conductivity detector or a flame ionization detector after conversion to a detectable form 25. Molecular hydrogen influences the ORP of a solution by acting as a reducing agent. Dissolved hydrogen lowers the ORP, indicating a more reductive environment. The Nernst equation captures this relationship, demonstrating that increased hydrogen concentrations result in a more negative ORP, reflecting greater reduction 26. While hydrogen activity can be equated to concentration in very dilute solutions, this relationship diverges at higher concentrations. To accurately apply the Nernst equation in such cases, the true activity of molecular hydrogen must be determined experimentally. ORP and hydrogen concentration may not always correlate, we present the ORP data, which may better represent the physicochemical effects of molecular hydrogen, as illustrated in Figure 3.

**Figure 9:**
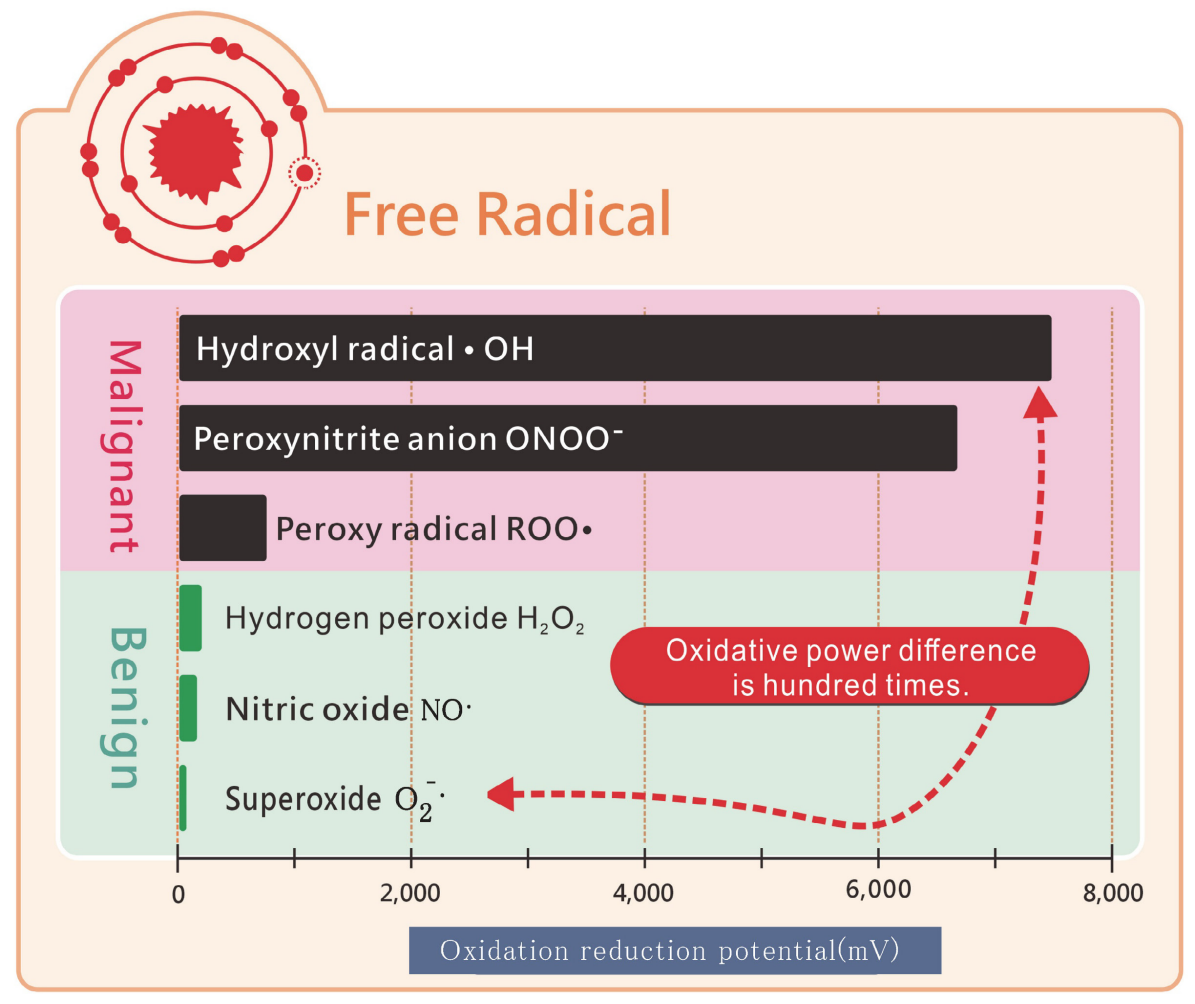
Molecular hydrogen eliminates toxic free radicals but not benign free radicals.

Molecular hydrogen can selectively target toxic free radicals. Functional benign free radicals, which are crucial for immunity against viruses and pathogens or for promoting blood vessel elasticity, do not require complete neutralization. While these benign free radicals have low oxidative capacity, malignant free radicals exhibit 100 times greater oxidative capacity (Figure 9). Molecular hydrogen, which is relatively mild, does not neutralize benign free radicals but focuses on toxic free radicals with super-high oxidizing power. Among the most toxic free radicals, ^●^OH**^-^** and molecular hydrogen promptly combine and react with it, generating water that can be easily excreted from the body 9.

### Anti-inflammatory effects of molecular hydrogen

CRP concentrations and the ESR are widely used blood test markers to assess inflammation in the body 27-29. Supplementation with molecular hydrogen has been shown to confer a protective effect by diminishing oxidative stress and suppressing the inflammatory response 30, 31. Additionally, it can regulate energy metabolism within the mitochondria 32. The results of a pharmacokinetic study using a swine model showed that in addition to hydrogen diffusing throughout the entire body after inhalation, molecular hydrogen is also transported throughout the body via the circulatory system, resulting in a comprehensive impact 33. Recent studies on molecular hydrogen have demonstrated noteworthy improvements in both patient and animal models, irrespective of the form of molecular hydrogen supplementation. For instance, obese mice exhibit enhanced fatty liver conditions after inhaling hydrogen gas or consuming HRW 34. In normal rats, inhaling hydrogen and drinking HRW effectively lowered visceral fat and blood lipid concentrations 10. Furthermore, in hypertensive rats, hydrogen inhalation resulted in a reduction in oxidative stress and the promotion of an anti-inflammatory response, leading to improvements in pulmonary hypertension 35, 36.

In mice experiencing acute pancreatitis, the inhalation of hydrogen was shown to be beneficial for reducing oxidative stress and inhibiting inflammatory responses 31. In mouse models, the consumption of HRW is effective at reducing chronic inflammation, improving symptoms of depression and anxiety, and relieving neuropathic pain 37, 38. The documented effects of molecular hydrogen supplementation in humans include a decrease in plasma low-density lipoprotein cholesterol (LDL-C) and free fatty acids along with an increase in extracellular superoxide dismutase after HRW consumption in patients with type 2 diabetes or impaired glucose tolerance 39. Moreover, HRW consumption resulted in a reduction in LDL-C and an improvement in high-density lipoprotein cholesterol (HDL-C), in addition to enhancements in cardiovascular and metabolic disease-related indicators, in patients with metabolic syndrome 40, 41. Molecular hydrogen supplementation alleviates inflammation in individuals with diabetes and heart disease 42-44, as well as in those with chronic obstructive pulmonary disease or asthma 19, 45, 46. There is a notable trend towards improved blood test indicators in patients with nonalcoholic fatty liver disease after eight weeks of HRW consumption 47. In addition to its anti-inflammatory effects, molecular hydrogen supplementation, whether through inhalation or ingestion of OSHCs, has also been associated with improved athletic performance and protection against fatigue and sports injuries 48, 49. Recent human research also highlights the potential of OSHC therapy for managing progressive fibrosing interstitial lung disease complicated by pneumonia. Following OSHCs treatment, a significant increase in the number of resting regulatory T cells was observed, along with a marked reduction in the number of Fas-positive helper T cells and cytotoxic T-cell subsets 50.

In summary, molecular hydrogen consumption has remarkable antioxidant and anti-inflammatory effects through specific neutralization of toxic free radicals. Importantly, these effects are achieved without disrupting the body's redox balance; instead, molecular hydrogen consumption modulates chronic inflammation and apoptosis, including a reduction in mitochondrial oxidative damage. Further evidence supports the ability of hydrogen gas to mitigate the side effects induced by chemotherapy and inhibit the growth of tumor cells, highlighting its potential therapeutic application in clinical practice 51, 52. Oral molecular hydrogen also serves as an effective antioxidant against exercise-induced ROS and can promote improvements in exercise performance in humans 53, 54. Consequently, future studies should explore the molecular mechanisms targeting redox-associated signaling pathways in chronic diseases. Additionally, the utilization of solid-form hydrogen compounds may represent a potential therapeutic strategy for the chemoprevention of chronic diseases in the future. A recent clinical study highlights the potential therapeutic effects of oral molecular hydrogen as a promising treatment for immunoglobulin G4-related pleuroparenchymal fibroelastosis (IgG4-PF-ILD) 50. The observational study result demonstrated that oral molecular hydrogen treatment had a regulatory effect on the immune system, particularly on T cell populations. Regulatory T cells (Tregs), which play a crucial role in aintaining immune tolerance and preventing autoimmunity, increased in number. This could indicate that the treatment is enhancing the body's ability to regulate immune responses and prevent excessive inflammation. The decrease in Fas^+^ helper T cells and cytotoxic T cells is also significant. In addition, a reduction in Fas^+^ T cells suggests a decrease in the propensity for these cells to undergo apoptosis or a shift in the balance of T cell subtypes towards those that are less prone to Fas-mediated cell death. These changes could reflect a modulation of the immune response, potentially making it more controlled and less prone to autoimmune reactions 50, 55.

The strength of this research is that it is the first to investigate the antioxidant and anti-inflammatory effects of a novel nutritional molecular hydrogen supplement in patients with chronic diseases. However, there are some limitations to this study. The sample size was small, and the treatment duration was short. Nevertheless, these preliminary data indicate that high-dose molecular hydrogen supplementation can induce antioxidative and anti-inflammatory effects, such as improvements in the ESR120, within just one week, and the effect persists for four weeks. Although trends were observed for the low and medium doses, the results were not statistically significant. Therefore, in future clinical studies, it is recommended to increase the number of patients and/or extend the treatment period. Additionally, the incorporation of a negative (placebo) control group is essential. Proinflammatory cytokines, which are mainly produced by activated macrophages, play a role in upregulating inflammatory reactions. Monitoring changes in interleukin-1, interleukin-6, tumor necrosis factor-α, and other significant oxidative and anti-inflammatory markers could provide valuable insights. Finally, gas chromatography may offer high accuracy and sensitivity, but it necessitates specialized equipment and expertise that are often not available in clinical settings.

The results of this study suggest that oral OSHCs supplementation may exert a notable anti-inflammatory effect on outpatients with chronic illnesses. Furthermore, these findings confirm that oral OSHC supplementation has the potential to alleviate symptoms associated with human diseases. We anticipate exploring additional molecular applications of OSHCs supplementation as an adjunct treatment for various diseases or dysfunctions in the future.

## Figures and Tables

**Table 1:** The demographic and baseline characteristics of the patients (n=30).

Characteristics	Dose: 1 capsule	Dose: 3 capsules	Dose: 6 capsules	*P-*value	Total
Patients (n)	10	10	10	--	30
Age, mean ± SD (years)	67.80±12.44	69.67±8.31	57.60±14.13	0.052	64.86±12.77
Gender	M: n=4 F: n=6	M: n=4 F: n=6	M: n=2 F: n=8	0.549	M: n=10 F: n= 20
ESR120	76.6±48.5	59.3±35.4	87.8±41.7	0.465	74.6±42.4
CRP (mg/dl)	2.7±5.2	0.2±0.1	1.3±2.5	0.463	1.5±3.4 31.1±20.1
BFI-T	35.6±17.2	34.5±22.4	23.2±20.0	0.152	
CSSD70	14.2±6.5	16.3±3.8	15.7±4.4	0.562	15.4±4.9

*: *p*-value<0.05; One-way ANOVA test was employed to compare values of the three groups for Age, ESR120, CRP, BFI-T, and CSSD70, and Chi-square test was used to compare the distribution of patients in different groups for Gender. **Abbreviations:** BFI-T, Brief Fatigue Inventory Taiwan; CRP, C-reactive protein; CSSD70, Control status scale for diabetes; ESR, Erythrocyte sedimentation rate; F, Female; M, Male.
